# Upregulation of microRNA-328-3p by hepatitis B virus contributes to THLE-2 cell injury by downregulating FOXO4

**DOI:** 10.1186/s12967-020-02299-8

**Published:** 2020-03-30

**Authors:** Xiaoyu Fu, Yi Ouyang, Juan Mo, Ronghua Li, Lei Fu, Shifang Peng

**Affiliations:** 1 Department of Infectious Diseases, Hunan Key Laboratory of Viral Hepatitis, Xiangya Hospital, Central South University, No.87 Xiangya Road, Changsha, 410008 Hunan China; 2 Department of Nuclear Medicine, Xiangya Hospital, Central South University, Changsha, Hunan China

**Keywords:** Hepatitis B virus, STAT3, miR-328-3p, FOXO4, THLE-2

## Abstract

**Background:**

Hepatitis B virus (HBV) remains a major cause of chronic hepatitis and hepatocellular carcinoma, and miRNAs play important roles in HBV pathogenesis. Our previous study has shown that miR-328-3p is upregulated in HBV-infected patients and serves as a potent predictor for the prognosis of HBV-related liver failure.

**Methods:**

Here, the role of miR-328-3p in modulating cell injury in HBV-infected liver cells THLE-2 was investigated in detail. MiR-328-3p expression was examined using qRT-PCR. The levels of pro-inflammatory cytokines were measured using ELISA. HBV RNA and HBV DNA levels were quantified. The interactions between STAT3 and miR-328-3p promoter as well as miR-328-3p and FOXO4 were analyzed using chromatin immunoprecipitation (CHIP) assay and luciferase reporter assay, respectively. THLE-2 cell injury was evaluated by examining cell viability and apoptosis.

**Results:**

HBV promoted expression of miR-328-3p through the STAT3 signal pathway and that increasingly expressed miR-328-3p downregulated its target FOXO4, leading to the promotion of cell injury in HBV-infected liver cells THLE-2.

**Conclusion:**

These data demonstrate that HBV-STAT3-miR-328-3p-FOXO4 regulation pathway may play an important role in the pathogenesis of HBV infection.

## Background

Hepatitis B virus (HBV) infection is a major public health problem and affects more than 400 million people worldwide [1, 2]. Therefore, a better understanding of the molecular mechanisms underlying the HBV pathogenesis is warranted. A most recent study demonstrated that HBV can regulate apoptosis of liver hepatocellular cells HepG2 via upregulating expression of certain microRNAs (miRNAs) [3], indicating the potential role of miRNAs in development of HBV-related liver disease.

miRNAs are small noncoding RNAs with 19–23 nucleotide in lengths and play an important regulatory role in various biological processes [4, 5]. Recent studies have highlighted the role of miRNAs in HBV pathogenesis [6–8]. Our previous study showed that serum miR-146a-5p, miR-122-3p and miR-328-3p levels were upregulated in patients with acute-on-chronic liver failure (ACLF) and chronic hepatitis B (CHB) compared with the chronic asymptomatic HBV carriers (ASC) [9]. Furthermore, the increased levels of these three miRNAs positively correlate with the severity of liver inflammation in ACLF patients and may be useful to predict the severity of HBV-associated ACLF [9]. Among these three miRNAs, miR-146a-5p and miR-328-3p could distinguish ACLF from non-ACLF (ASC and CHB) with high values of specificity and sensitivity [9]. We have also previously demonstrated that miR-146a-5p enhances HBV replication through autophagy to promote aggravation of CHB [10]. MiR-146a has been shown to directly target CXCR4 [11], a gene involved in the advanced liver disease that is associated with hepatitis C virus or HBV [12]. Intriguingly, upregulation of miR-146a induced by HBV X protein (HBx) through NF-κB-mediated enhancement of miR-146a promoter activity contributes to hepatitis development [8]. However, whether HBV can regulate miR-328-3p and the underlying mechanism remain unclear.

Recent studies have suggested that HBV can induce activation of STAT3 (signal transducer and activator of transcription 3) in hepatocytes to foster its own replication but also to prevent apoptosis of infected cells [13, 14]. Furthermore, knockout of hepatitis B surface antigen (HBsAg) inhibits STAT3 signaling, while overexpression of HBsAg induces a substantial accumulation of STAT3 phosphorylation [15]. STAT3 has been shown to play important roles in liver inflammatory responses [16]. More importantly, our bioinformatics analysis revealed that STAT3 was identified as a predicted transcription factor for miR-328-3p (LASAGNA-Search 2.0 and TransmiR v2.0 database). Therefore, we speculated that HBV may promote transcription of miR-328-3p via activating STAT3 signaling.

miRNAs are post-transcriptional regulators that bind to the 3′-untranslated region (3′-UTR) of the target gene messenger RNA [17, 18]. Our bioinformatics analysis demonstrated that FOXO4 was a putative target of miR-328-3p by harboring a miR-328-3p binding sequence in the 3′-UTR of its mRNA (Targetscan). FOXO4 has been identified as an endogenous inhibitor of NF-κB [19] which mediates induction of pro-inflammatory cytokines. In addition, we found that FOXO4 expression was down-regulated in HBV-infected human primary hepatocytes by analysis of the GEO database (GSE72068). Therefore, we hypothesized that miR-328-3p may promote hepatocyte injury by down-regulating FOXO4 to promote cellular inflammatory response.

In the current study, we evaluated in detail the roles of miR-328-3p in modulating cell injury in HBV-infected liver cells THLE-2. Furthermore, the putative mechanisms of the HBV-STAT3-miR-328-3p-FOXO4 regulatory axis in this process were also explored.

## Materials and methods

### Human samples

Patients with acute-on-chronic liver failure (ACLF, n = 25, 19 male, mean age: 46.7 ± 10.1 years), chronic hepatitis B (CHB, n = 25, 17 male, mean age: 41.3 ± 11.5 years), chronic asymptomatic HBV carriers (ASC, n = 25, 18 female, mean age: 40.5 ± 10.8 years), and healthy volunteers as normal controls (NC, n = 25, 14 male, mean age: 40.5 ± 11.3 years) were enrolled from the Xiangya Hospital, from October 2016 to September 2017. ASC, CHB, and ACLF were diagnosed according to the Guideline of Prevention and Treatment for Chronic Hepatitis B (2015 Update) [20]. The diagnosis of ACLF was based on the following criteria: Acute hepatic insult manifested as jaundice (serum bilirubin ≥ 5 mg/dL or 85 µmol/L) and coagulopathy [international normalized ratio (INR) > 1.5 or prothrombin activity < 40%], complicated within 4 weeks by ascites and/or encephalopathy in a patient with CHB. CHB is defined as chronic HBV infection with clinical evidences of liver diseases such as biochemical, virological and histological features together with exclusion of other causes. Patients with CHB can be divided into HBeAg-positive and HBeAg negative. ASC is defined as HBsAg-positive, anti-HBe-positive with persistent normal serum alanine aminotransferase (ALT) and HBV-DNA < 2000 IU/mL, and no evidence of liver injury [21]. Patients exhibiting the following were excluded from the study: pregnancy, drug-induced liver injury, alcoholic liver disease, acute fatty liver, autoimmune liver disease, hepatolenticular degeneration, and liver failure after transplantation. The whole blood samples were collected from individuals and immediately centrifuged at 3000 rpm for 10 min at 4 °C. The resulting supernatant was serum that was stored at − 80 °C for subsequent experiments. All the experimental procedures were approved by the Ethics Committee of the Xiangya Hospital. Written informed consent was obtained from each participant.

### THLE-2 cell culture

The human liver THLE-2 cells were purchased from American Type Culture Collection (ATCC, Manassas, VA, USA) and cultured in Dulbecco’s modified Eagles medium (DMEM)/F12 (Gibco) supplemented with 10% fetal bovine serum (FBS, Gibco) at 37 °C in a humidified atmosphere containing 95% air and 5% CO_2_.

### Cell transfection and cell treatment

The HBV replication plasmid pHBV1.3 which contained 1.3 copies of the HBV genome was obtained from BioVector (Beijing, China). The plasmids expressing the four proteins of HBV (surface antigen (HBsAg), core protein (HBcAg), and DNA polymerase protein (HBp), and HBV X protein (HBx)) were cloned using PCR from pHBV1.3, generating pHBs, pHBc, pHBp, and pHBx, respectively. The pcDNA3.1 (Invitrogen) was used as the empty control vector. Human THLE-2 cells were transfected with pHBV1.3, pHBs, pHBc, pHBp, and pHBx using 1 μL of Lipofectamine 2000 (Invitrogen) according to the manufacturer’s instructions.

Hsa-miR-328-3p mimic, hsa-miR-328-3p inhibitor, mimic negative control (NC), and inhibitor NC were purchased from Applied Biological Materials Inc. (Richmond, BC, Canada; 50 nM) were transfected into THLE-2 cells using 1 μL of Lipofectamine™ RNAiMAX Transfection Reagent (Invitrogen) according to the manufacturer’s instructions.

To overexpress FOXO4, The full-length FOXO4 cDNA fragment was cloned into pcDNA 3.1 vector, generating pcDNA3.1-FOXO4, with an empty pcDNA3.1 as a control. To knockdown FOXO4, si-FOXO4 [sense 5′-UCUCACCUCUUCCCAUUCC(dTdT)-3′, antisense 5′-GGAAUGGGAAGAGGUGAGA(dTdT)-3′] and its scramble siRNA control (si-Ctrl) were obtained from GenePharma (Shanghai, China). These plasmids (60 nM) were transfected into THLE-2 cells using 1 μL of Lipofectamine™ RNAiMAX Transfection Reagent (Invitrogen) and the knockdown efficiency was determined by qRT-PCR at 48 h post-transfection.

### Detection of miR-328-3p expression

Total RNA was extracted from human serum samples or THLE-2 cells using TRIzol reagent (Invitrogen). RNA was quantified and then reverse transcribed into cDNAs using the TaqMan miRNA Reverse Transcription kit (Applied Biosystems, Waltham, MA, USA). The cDNA templates were amplified through real-time quantitative PCR (qRT-PCR) using SYBR^®^ Premix Ex Taq™ (Takara, Dalian, China) with a StepOne-plus Real-Time PCR System (Applied Biosystems). Data are presented as relative quantification based on the 2^−ΔΔCt^ method: ΔCt = Ct gene of interest − Ct internal control, while ΔΔCt = (Ct gene of interest − Ct internal control) sample − (Ct gene of interest − Ct internal control) control. The expression of miR-328-3p was normalized to the artificial miRNA Spike cel-miR-39 (for serum samples) or the internal control U6 snRNA (for cells). The primers used in this study was as follows: miR-328-3p-F, 5′-TGCGGCTGGCCCTCTCTGCCC-3′, miR-328-3p-R, 5′ CCAGTGCAGGGTCCGAGGT-3′ [22]; cel-miR-39-F, 5′-TCACCGGGTGTAAATCAGCTTG-3′ [23], cel-miR-39-R, universal primer (QIAGEN, Valencia, CA, USA); U6-F, 5′-TGCGGGTGCTCGCTTCGGCAGC-3′ U6-R, 5′-CCAGTGCAGGGTCCGAGGT-3′ [22].

### Quantification of HBV RNA and HBV DNA

Quantification of HBV RNA in THLE-2 cells was performed as previously described [24]. HBV RNA was isolated using the EasyPure Viral RNA Kit (TransGen Biotech, Beijing, China) and reverse transcribed using RevertAid First Strand DNA Synthesis Kit (Thermo Fisher Scientific, Waltham, MA, USA). The levels of HBV RNA were detected by quantitative real-time PCR with a SYBR Green or TaqMan probe method using LightCycler 480 II Real-time PCR Detection System (Roche, Mannheim, Germany).

The level of HBV DNA was quantified by artus HBV PCR Kits CE (QIAGEN) according to the manufacturer’s instructions.

### Enzyme-linked immunosorbent assay (ELISA)

The pro-inflammatory cytokines including tumor necrosis factor-alpha (TNF-α), interleukin (IL)-6, IL-8, IL-12, and IL-18 in human sera or THLE-2 cell supernatants were measured using human Inflammatory Cytokines Multi-Analyte ELISArray™ Kit (MEH-004A, QIAGEN) according to the manufacturer’s instructions.

Levels of HBsAg and HBeAg in THLE-2 cell supernatants were measured using their commercial ELISA kits (LifeSpan BioSciences) following the manufacturers’ protocols.

### Western blot

Total protein was extracted in ice-cold radioimmunoprecipitation assay lysis buffer (Beyotime, Shanghai, China). The protein concentrations were determined using a BCA Protein Assay Kit (Beyotime). The protein from cell lysates was separated by 10% sodium dodecyl sulfate–polyacrylamide gel electrophoresis (SDS-PAGE) gels and transferred onto polyvinylidene fluoride (PVDF) membranes (Millipore Corp., Billerica, MA, USA). After being blocked with 5% skim milk, the membranes were incubated with primary antibodies against FOXO4 (1:1000), STAT3 (1:1000), phosphorylated (p)-STAT3 (1:2000), IκB-α (1:1000), p65 (1:1000), and p-p65 (1:1000) overnight at 4 °C. All these primary antibodies were purchased from Cell Signaling Technology Inc., Danvers, MA, USA. The membranes were then washed with TBST and incubated with secondary antibodies horseradish peroxidase (HRP)-conjugated secondary antibodies (Cell Signaling Technology Inc.) at room temperature for 2 h. The protein levels were quantified using enhanced chemiluminescence (Thermo Scientific). Image-Pro Plus 6.0 software was used to analyze the band intensity. β-actin served as the loading control.

### Chromatin immunoprecipitation (CHIP)

CHIP assay was performed to analyze the interaction between STAT3 and miR-328-3p promoter using Simple ChIP Enzymatic Chromatin IP Kit (Cell Signaling Technology). Briefly, cells were cross-linked with 1% formaldehyde, harvested, and then incubated on ice for 10 min in lysis buffer. The lysates were sonicated to shear DNA. Subsequently, the sheared chromatin was incubated with anti-STAT3 or normal serum IgG (Cell Signaling Technology) overnight at 4 °C. Then, protein G beads were added to the mixtures for 2 h of incubation. The antibody-bound protein/DNA complexes were washed and eluted from the beads. After reversing crosslinks, DNA was purified and subjected to qRT-PCR analysis using promoter-specific primers (miR-328-3p promoter-F: TGTCAAGGTTCAGCGATGCT; miR-328-3p promoter-R: CCTTCTTCCTGCAGTCCCTG). An aliquot of chromatin that was not incubated with an antibody was used as the input control sample.

### Cell proliferation assay

The 3-(4,5-dimethylthiazol-2-yl)-2,5-diphenyltetrazolium bromide (MTT) assay was performed to analyze the cell growth at different time points in THLE-2 cells. After different treatment, cells were plated into 96-well plates at a density of 1500–2000 cells/well for 24 h of incubation. Then MTT (Sigma; 20 μL; 5 mg/mL) was added into each well. After 4 h of incubation, the supernatant was discarded carefully and DMSO (Sigma; 150 μL) was added to dissolve the formazan product for 10 min. The optical density (OD) at 490 nm was measured by a microplate reader (Multiskan Mk3, Thermo Labsystems, Finland).

### Cell apoptosis assay

An annexin V-fluorescein isothiocyanate (FITC)/propidium iodide (PI) cell apoptosis kit was used to qualify the cell apoptosis in THLE-2 cells. After the designated treatment, cells were harvested, washed twice with PBS, and re-suspended in the staining buffer provided in the kit. After this, Annexin V-FITC (5 µL) and PI (5 µL) were mixed with the cells for 10 min at room temperature in the dark. Finally, the apoptotic rates were detected using BD FACSAria flow cytometry (BD Biosciences, San Jose, CA, USA).

### Luciferase reporter assay

The 3′-untranslated region (UTR) of FOXO4 containing the predicted wild-type (WT) binding sites of miR-328-3p or mutated miR-328-3p binding sites (MUT) were amplified by PCR and cloned into a pGL3 vector, termed as FOXO4-WT, and FOXO4-MUT. For luciferase assay, cells were co-transfected with FOXO4-WT or FOXO4-MUT, and miR-328-3p mimic or negative control miRNA mimic (mimic NC) by Lipofectamine 2000 (Invitrogen). At 24 h post transfection, the luciferase activities were analyzed using a luciferase reporter assay system (Promega Corporation, Fitchburg, WI, USA).

### Statistical analysis

All statistical analyses were performed using SPSS version 16.0 (SPSS, Inc., Chicago, USA). The unpaired Student’s *t*-test and one-way analysis of variance (ANOVA) were used to analyze differences between groups. Values are presented as the mean ± standard deviation (SD) from three independent experiments. P < 0.05 was considered to indicate a statistically significant difference.

## Results

### HB patients exhibit increased expression of miR-328-3p and pro-inflammatory cytokines

**Fig. 1:**
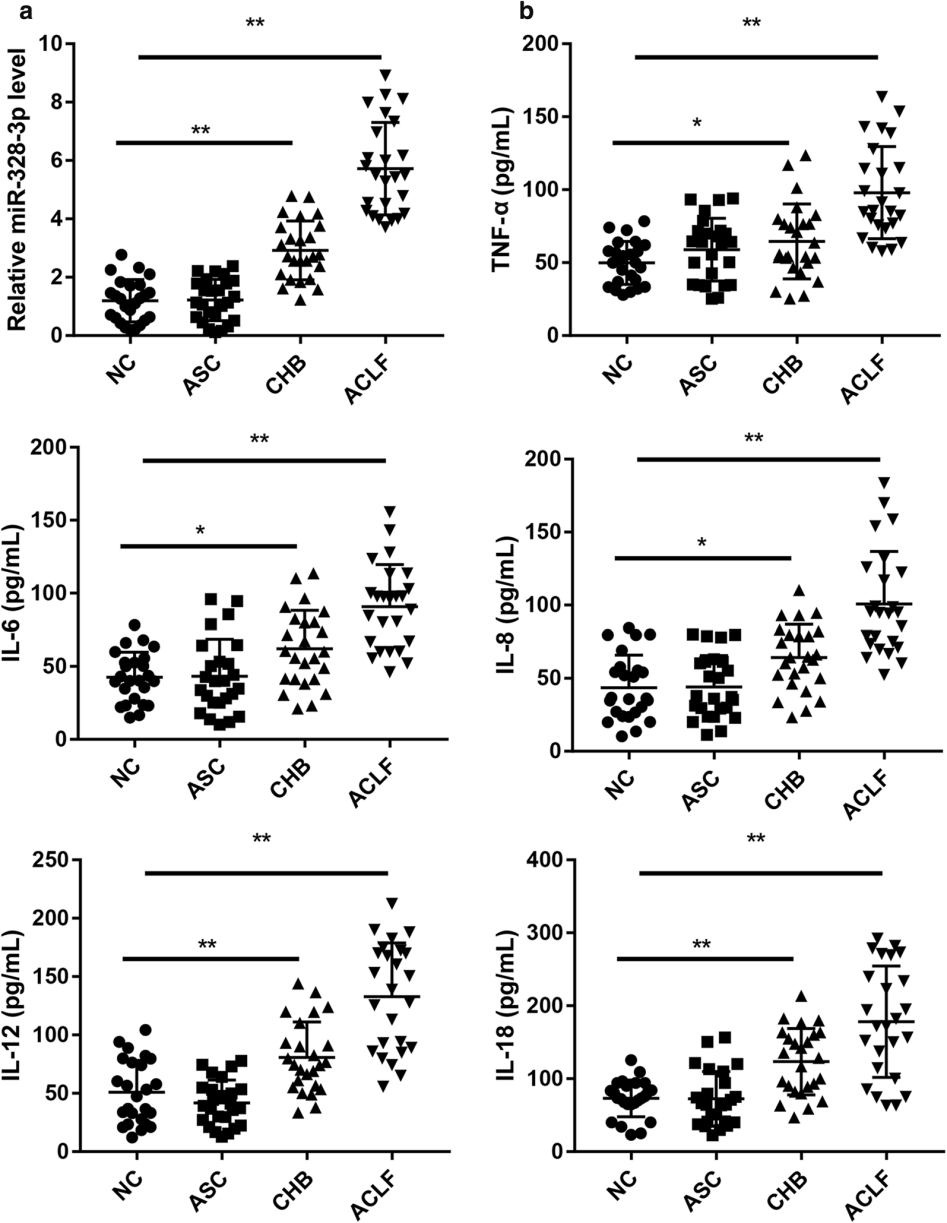
HB patients exhibit increased expression of miR-328-3p and pro-inflammatory cytokines. Relative miR-328-3p expression (**a**) and levels of pro-inflammatory cytokines including TNF-α, IL-6, IL-8, IL-12, IL-18 (**b**) in sera from patients with ASC, CHB, ACLF, and their normal controls. N = 25 in each group. *P < 0.05, **P < 0.01 vs. NC

Compared with the NC subjects, the CHB and ACLF patients exhibited significantly higher serum miR-328-3p expression (Fig. 1a), accompanied with higher serum pro-inflammatory cytokines including TNF-α, IL-6, IL-8, IL-12, IL-18 (Fig. 1b). These results indicate that miR-328-3p expression may correlate with enhanced inflammation in HB patients caused by HBV infection.

### HBV/HBc/HBx increases miR-328-3p expression and STAT3 phosphorylation, whereas decreases FOXO4 expression

**Fig. 2:**
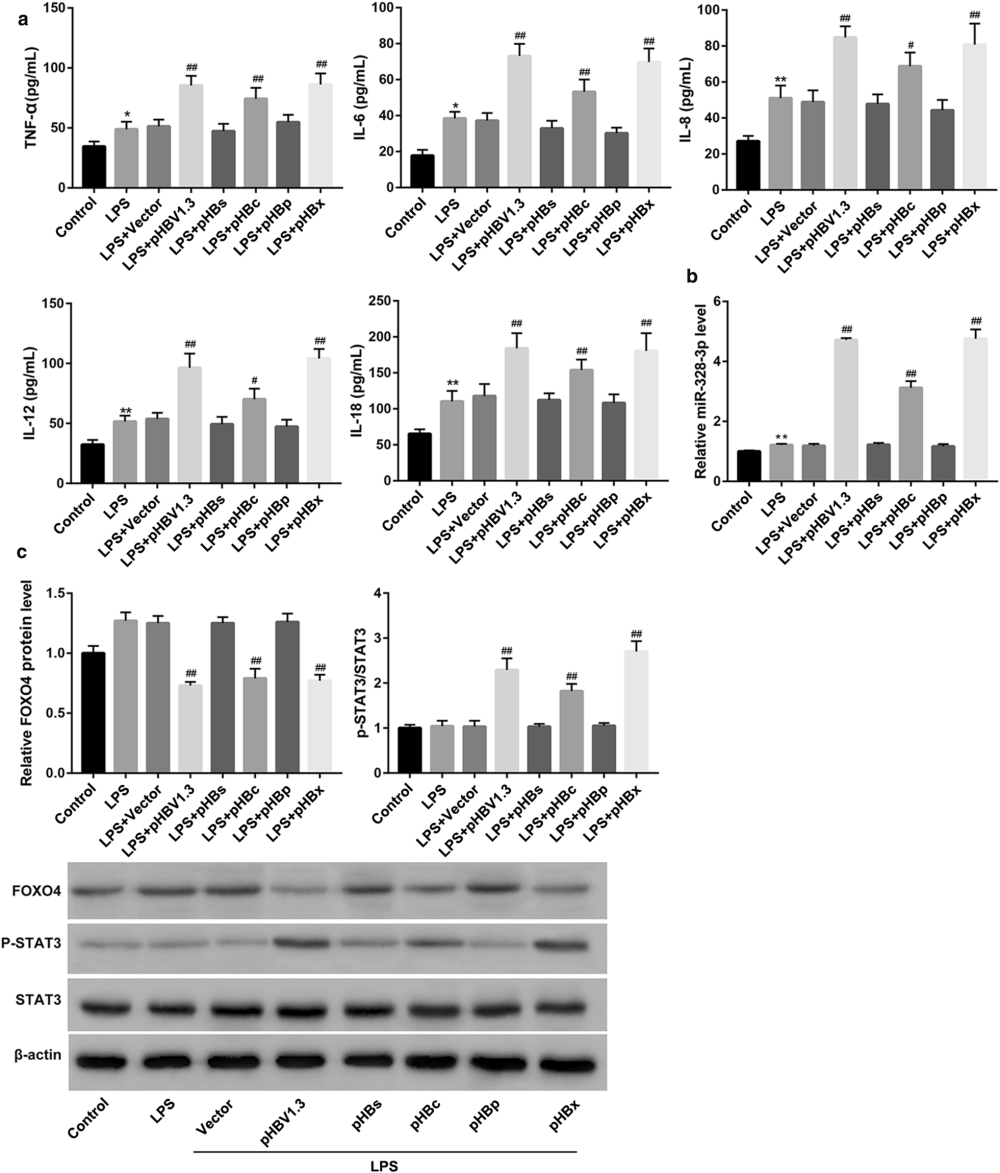
Effect of HBV/HBs/HBc/HBp/HBx on pro-inflammatory cytokines, miR-328-3p expression, STAT3 phosphorylation, and FOXO4 protein expression. THLE-2 cells were transfected with pHBV1.3 (HBV replication plasmid), pHBs (expressing HBsAg), pHBc (expressing HBcAg), pHBp (expressing DNA polymerase protein), pHBx (expressing HBx), and empty pcDNA3.1 vector (control), under LPS stimulation (1 μg/mL, 24 h). **a** ELISA was performed to detect levels of TNF-α, IL-6, IL-8, IL-12, and IL-18. **b** qRT-PCR was conducted to examine miR-328-3p expression. **c** Western blot was performed to measure protein expression of FOXO4 and STAT3, and phosphorylated (p)-STAT3. Values are presented as the mean ± SD (n = 3). *P < 0.05, **P < 0.01 vs. NC. ^#^P < 0.05, ^##^P < 0.01 vs. LPS + vector

To investigate the effect of HBV and its four proteins (HBc, HBx, HBs, HBp) on miR-328-3p and FOXO4 expression as well as STAT3 phosphorylation, we transfected THLE-2 cells with pHBV1.3, pHBc, pHBx, pHBs, and pHBp to overexpress them respectively. Data revealed that overexpression of HBV, HBc, and HBx significantly enhanced the lipopolysaccharide (LPS)-induced elevation of pro-inflammatory cytokines (Fig. 2a) and miR-328-3p expression (Fig. 2b). Furthermore, overexpression of HBV, HBc, and HBx notably increased STAT3 phosphorylation, but decreased FOXO4 protein expression, under LPS stimulation (Fig. 2c). However, HBs and HBp overexpression had no significant effect on levels of pro-inflammatory cytokines (Fig. 2a), miR-328-3p expression (Fig. 2b), FOXO4 protein expression and STAT3 phosphorylation (Fig. 2c) under LPS stimulation.

### STAT3 activates miR-328-3p transcription and mediates the HBV/HBc/HBx-induced upregulation of miR-328-3p

**Fig. 3:**
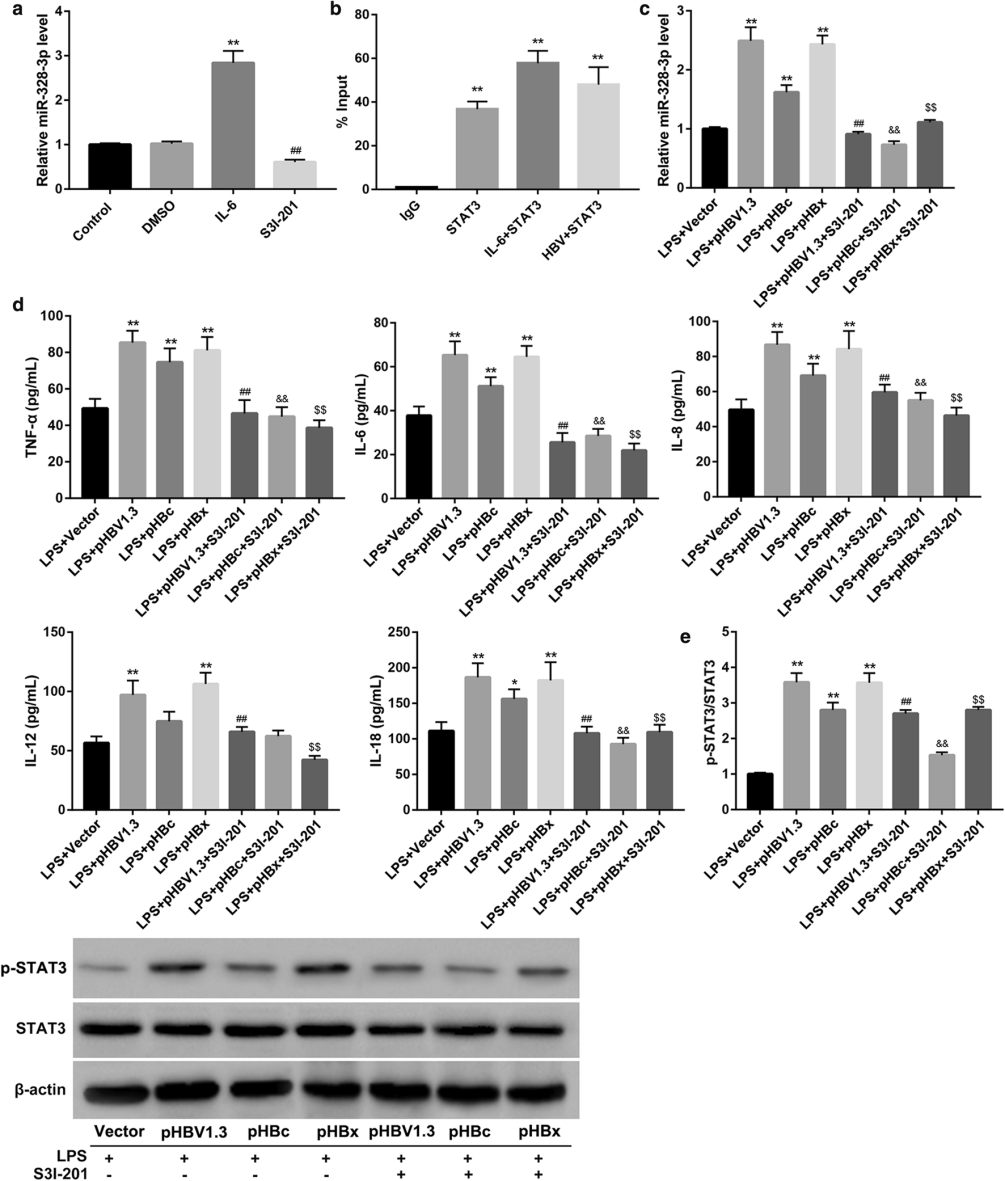
STAT3 activates miR-328-3p transcription and mediates the HBV/HBc/HBx-induced upregulation of miR-328-3p. **a** THLE-2 cells were cultured with recombinant human IL-6 protein (200 ng/mL), S3I-201 (50 μM), or DMSO (vehicle control) for 24 h, followed by qRT-PCR analysis of miR-328-3p expression. **b** Resultant ChIP DNA was amplified by qRT-PCR. CHIP-qPCR assay confirmed the direct binding of STAT3 to the miR-328 promoter. IgG served as a negative control. IL-6 was used to activate STAT3. **c**–**e** THLE-2 cells were transfected with pHBV1.3, pHBc, pHBx and empty pcDNA3.1 vector (control), followed by S3I-201 (50 μM, 24 h) or not, under LPS stimulation (1 μg/mL, 24 h). Then **c** qRT-PCR was conducted to examine miR-328-3p expression. **d** ELISA was performed to detect levels of TNF-α, IL-6, IL-8, IL-12, and IL-18. **e** Western blot was performed to measure protein expression of STAT3, and p-STAT3. Values are presented as the mean ± SD (n = 3). *P < 0.05, **P < 0.01 vs. LPS + Vector. ^##^P < 0.01 vs. LPS + pHBV1.3. ^&&^P < 0.01 vs. LPS + pHBc. ^$$^P < 0.01 vs. LPS + pHBx

We further investigated whether STAT3 can transcriptionally activate miR-328-3p. Data demonstrated that IL-6 (a STAT3 activator) significantly increased miR-328-3p expression, whereas S3I-201 (a STAT3 inhibitor) notably decreased miR-328-3p expression (Fig. 3a). CHIP assay further confirmed the direct binding of STAT3 to the miR-328-3p promoter (Fig. 3b). Furthermore, compared with the control THLE-2 cells, IL-6- or HBV- stimulated THLE-2 cells exhibited significantly increased binding capacities between of STAT3 to the miR-328-3p promoter (Fig. 3b). We, therefore, tested whether STAT3 mediates HBV/HBc/HBx-induced upregulation of miR-328-3p expression. The results showed that S3I-201 effectively abrogated the pHBV1.3, pHBc, pHBx-mediated upregulation of miR-328-3p expression (Fig. 3c), pro-inflammatory cytokines (Fig. 3d), and STAT3 phosphorylation (Fig. 3e). Overall, these data suggest that STAT3 activates miR-328-3p transcription and mediates the HBV/HBc/HBx-induced upregulation of miR-328-3p.

### MiR-328-3p inhibitor suppresses HBV activity and attenuates the HBV-mediated cell injury

**Fig. 4:**
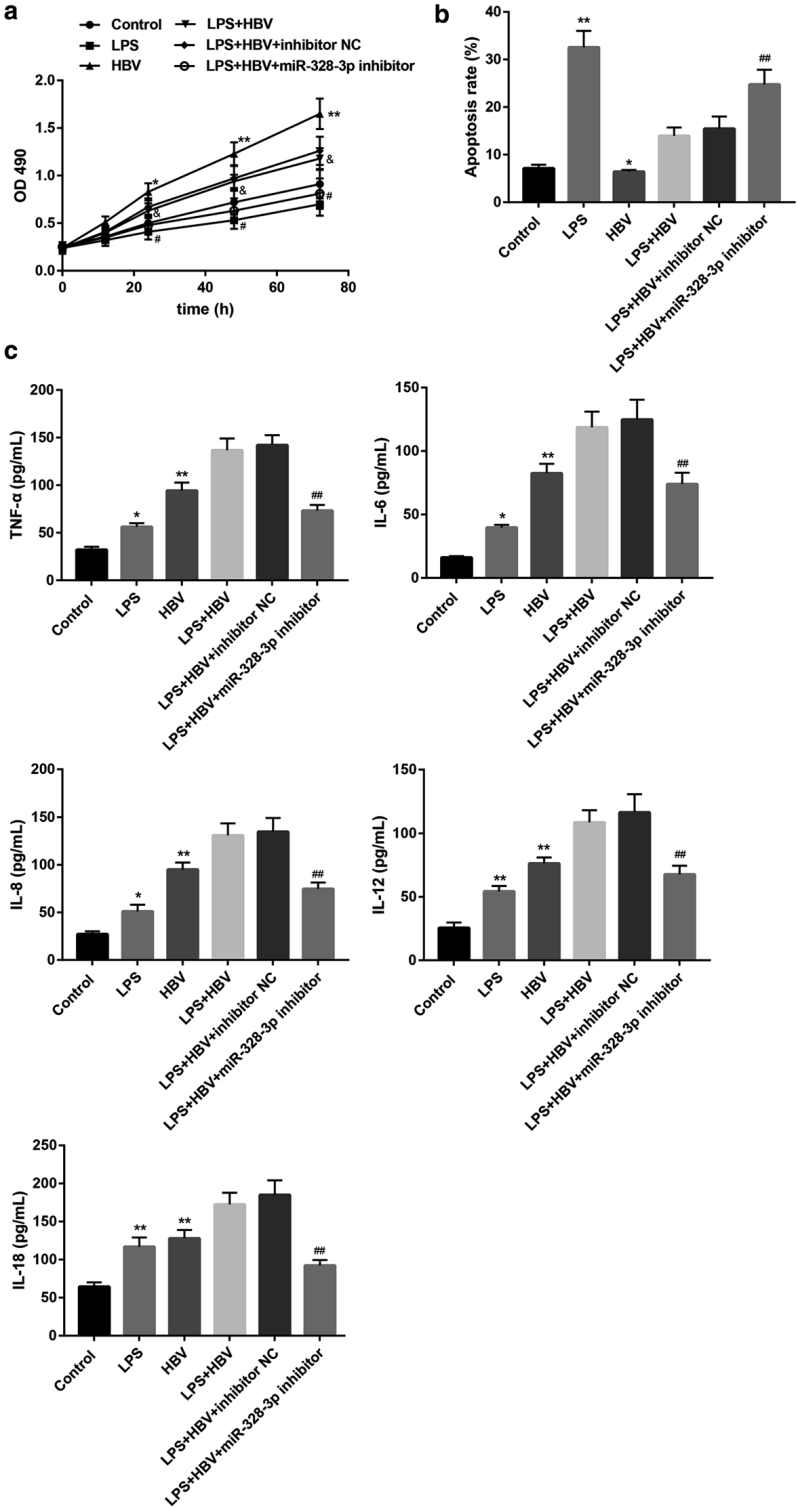
miR-328-3p inhibitor attenuates the HBV-mediated cell injury. THLE-2 cells were transfected with miR-328-3p inhibitor or inhibitor negative control (NC), under stimulation with LPS (1 μg/mL, 24 h) or/and HBV. Then **a** cell proliferation at different time points was examined by MTT assay. **b** Cell apoptosis was examined by flow cytometry and the apoptosis rate was shown. **c** Levels of TNF-α, IL-6, IL-8, IL-12, and IL-18 were measured by ELISA. Values are presented as the mean ± SD (n = 3). *P < 0.05, **P < 0.01 vs. Control. ^#^P < 0.05, ^##^P < 0.01 vs. LPS + HBV + inhibitor NC

To investigate the effect of miR-328-3p on the HBV-induced THLE-2 cell injury, we transfected THLE-2 cells with miR-328-3p inhibitor, followed by LPS and HBV stimulation. LPS stimulation significantly inhibited proliferation and promoted apoptosis, whereas HBV promoted proliferation and suppressed apoptosis (Fig. 4a, b and Additional file 1: Figure S1). Furthermore, both LPS and HBV enhanced levels of pro-inflammatory cytokines (Fig. 4c). Importantly, miR-328-3p inhibitor abolished the HBV-mediated proliferation promotion (Fig. 4a), apoptosis inhibition (Fig. 4b), and pro-inflammatory cytokines elevation (Fig. 4c).

**Fig. 5:**
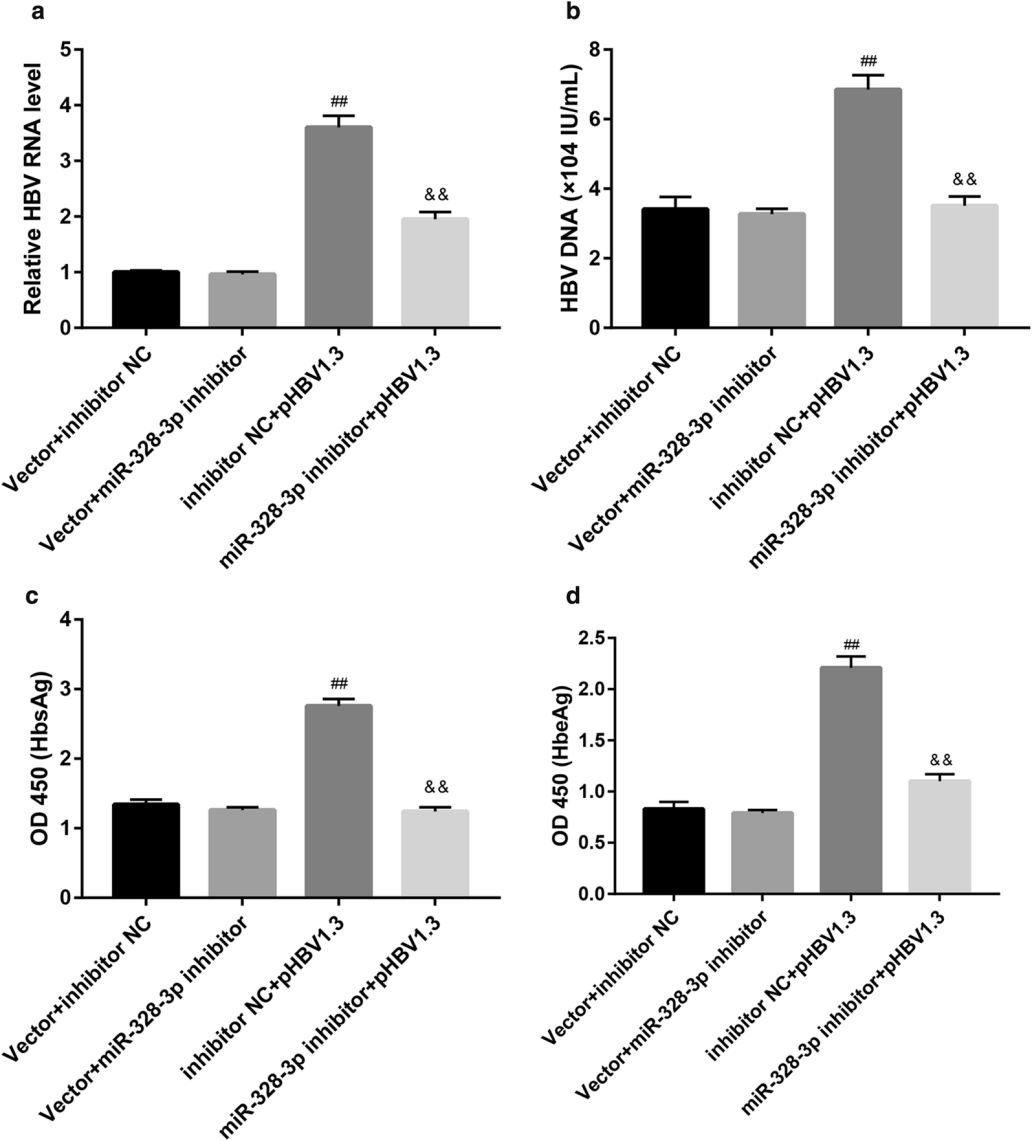
miR-328-3p inhibitor suppresses pHBV1.3-induced HBV activity. THLE-2 cells were co-transfected with pHBV1.3 or empty pcDNA3.1 vector (control), and miR-328-3p inhibitor or inhibitor negative control (NC). **a** HBV RNA and **b** HBV DNA were qualified. **c**, **d** The OD 450 absorbance values were obtained by ELISA for determining levels of HBsAg and HBeAg in THLE-2 cell supernatants. Values are presented as the mean ± SD (n = 3). ^##^P < 0.01 vs. Vector + inhibitor NC. ^&&^P < 0.01 vs. inhibitor NC + pHBV1.3

To further explore the effect of miR-328-3p on HBV activity in THLE-2 cells, we transfected THLE-2 cells with pHBV1.3 and miR-328-3p inhibitor, both alone and in combination. Transfection with pHBV1.3 elevated both RNA (Fig. 5a) and DNA levels of HBV (Fig. 5b), and levels of HBsAg (Fig. 5c) and HBeAg (Fig. 5d). Of note, miR-328-3p inhibitor exerted the opposite effect and abrogated the pHBV1.3-induced elevation in both RNA (Fig. 5a) and DNA levels of HBV (Fig. 5b), and levels of HBsAg (Fig. 5c) and HBeAg (Fig. 5d). Altogether, these data indicate that miR-328-3p inhibitor suppresses HBV activity.

### MiR-328-3p targets FOXO4, and activates downstream NF-κB pathway

**Fig. 6:**
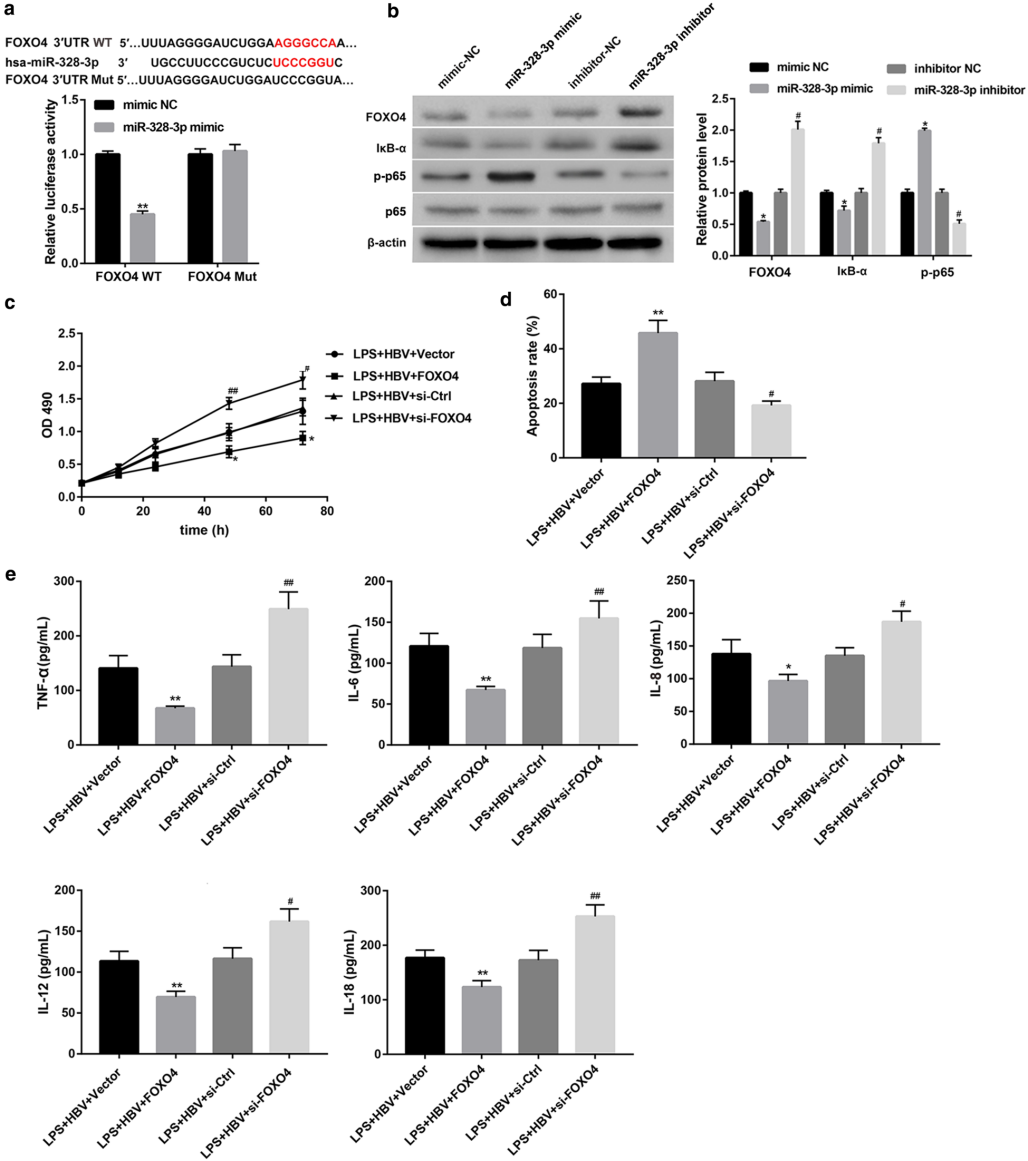
FOXO4 is a target for miR-328-3p and attenuates cell injury under LPS and HBV stimulation. **a** Results of luciferase activity assay showed that 3′UTR of FOXO4 was directly targeted by miR-328-3p. **P < 0.01 vs. mimic NC + FOXO4 WT. **b** Western blot analysis of protein expression of FOXO4, IκB-α, p65, and p-p65 in THLE-2 cells transfected with miR-328-3p mimic, miR-328-3p inhibitor, or corresponding controls. *P < 0.05 vs. the mimic NC group, ^#^P < 0.05 vs. the inhibitor NC group. **c** Cell proliferation detectecd by MTT assay, **d** cell apoptosis rate detected by flow cytometry, and **e** levels of TNF-α, IL-6, IL-8, IL-12, and IL-18 detected by ELISA in THLE-2 cells transfected with pcDNA3.1-FOXO4, si-FOXO4, or corresponding controls, under LPS (1 μg/mL, 24 h) and HBV stimulation. Values are presented as the mean ± SD (n = 3). *P < 0.05, **P < 0.01 vs. LPS + HBV + Vector, ^#^P < 0.05, ^##^P < 0.01 vs. LPS + HBV + si-Ctrl

Luciferase reporter assay showed a decreased luciferase activity in cells co-transfected with miR-328-3p mimics and WT FOXO4 3′UTR luciferase reporter plasmids (Fig. 6a), indicating that 3′UTR of FOXO4 is directly targeted by miR-328-3p. Furthermore, miR-328 mimic inhibited FOXO4 protein expression, whereas miR-328-2p inhibitor increased FOXO4 protein expression (Fig. 6b), further verifying the negative regulation of FOXO4 by miR-328-3p. FOXO4 is an endogenous inhibitor of NF-κB [19], which was known to play a central role in liver inflammatory responses [16]. Importantly, the results showed that miR-328 mimic decreased IκB-α protein expression and increased p65 phosphorylation, whereas miR-328 inhibitor caused the opposite effect (Fig. 6b). Taken together, these results suggested that miR-328-3p targets FOXO4 and activates its downstream NF-κB pathway.

### FOXO4 suppresses HBV activity and attenuates cell injury under LPS and HBV stimulation

**Fig. 7:**
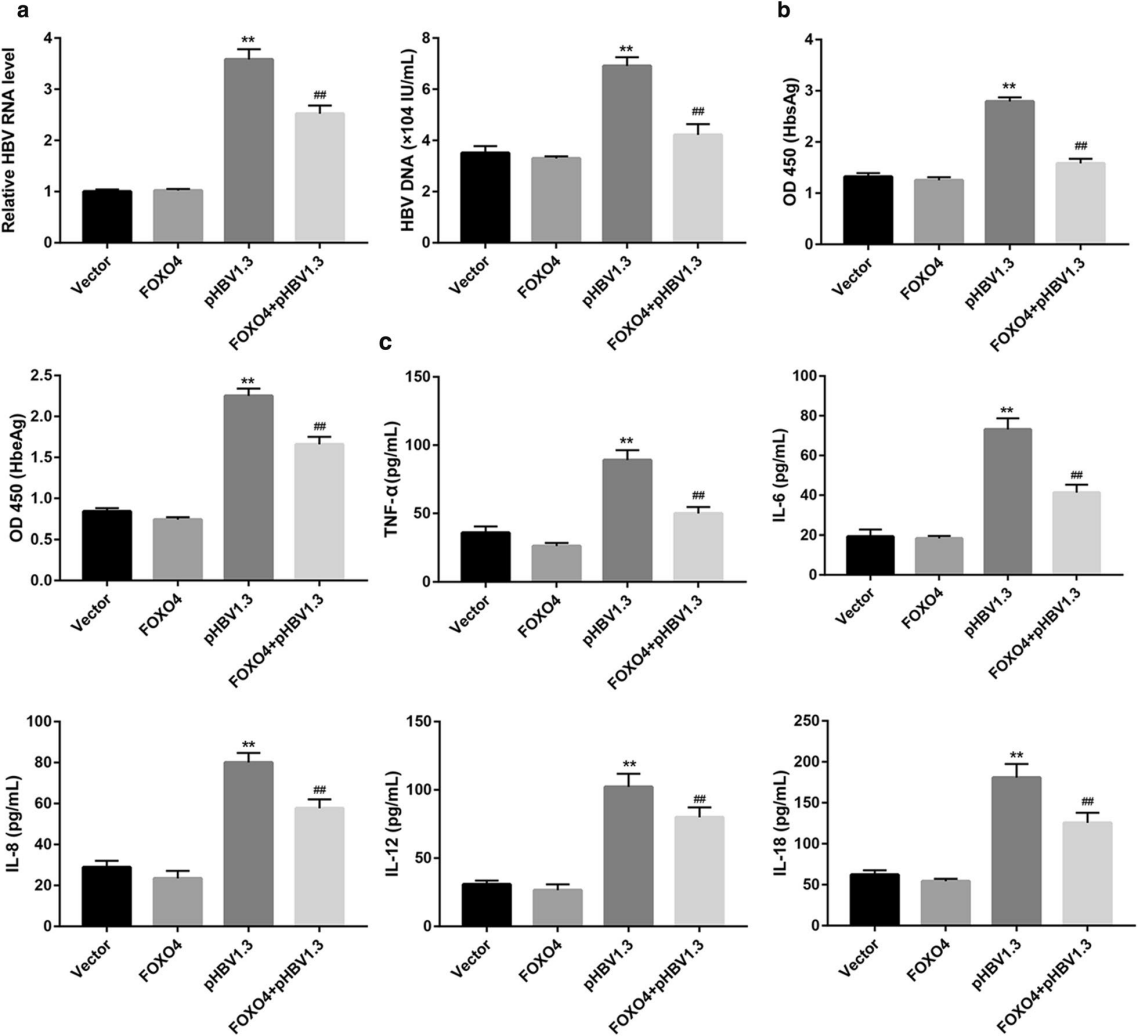
FOXO4 suppresses pHBV1.3-induced HBV activity. THLE-2 cells were transfected with pHBV1.3 and pcDNA3.1-FOXO4, both alone and in combination. **a** HBV RNA and **b** HBV DNA were qualified. **c** The OD 450 absorbance values were obtained by ELISA for determining levels of HBsAg and HBeAg in THLE-2 cell supernatants. Values are presented as the mean ± SD (n = 3). **P < 0.01 vs. Vector. ^##^P < 0.01 vs. pHBV1.3

To explore the effect of FOXO4 expression on cell injury, we overexpressed and silenced FOXO4 in THLE-2 cells, followed by LPS and HBV stimulation. Successful overexpression and knockdown were confirmed by qRT-PCR (Additional file 2: Figure S2A). FOXO4 overexpression inhibited cell proliferation (Fig. 6c), promoted cell apoptosis (Fig. 6d and Additional file 2: Figure S2B), and decreased pro-inflammatory cytokines (Fig. 6e), under stimulation with LPS and HBV. In contrast, FOXO4 knockdown exerted the opposite effect (Fig. 6c–e). These data suggest that FOXO4 attenuates cell injury. We next explored the effect of FOXO4 on HBV activity in THLE-2 cells. FOXO4 overexpression significantly abrogated the pHBV1.3-induced elevation in both RNA and DNA levels of HBV (Fig. 7a), and levels of HBsAg and HBeAg (Fig. 7b), and pro-inflammatory cytokines (Fig. 7c). Overall, these data indicate that FOXO4 overexpression suppresses HBV activity.

### FOXO4 overexpression abrogates the miR-328-3p-mediated cell injury

**Fig. 8:**
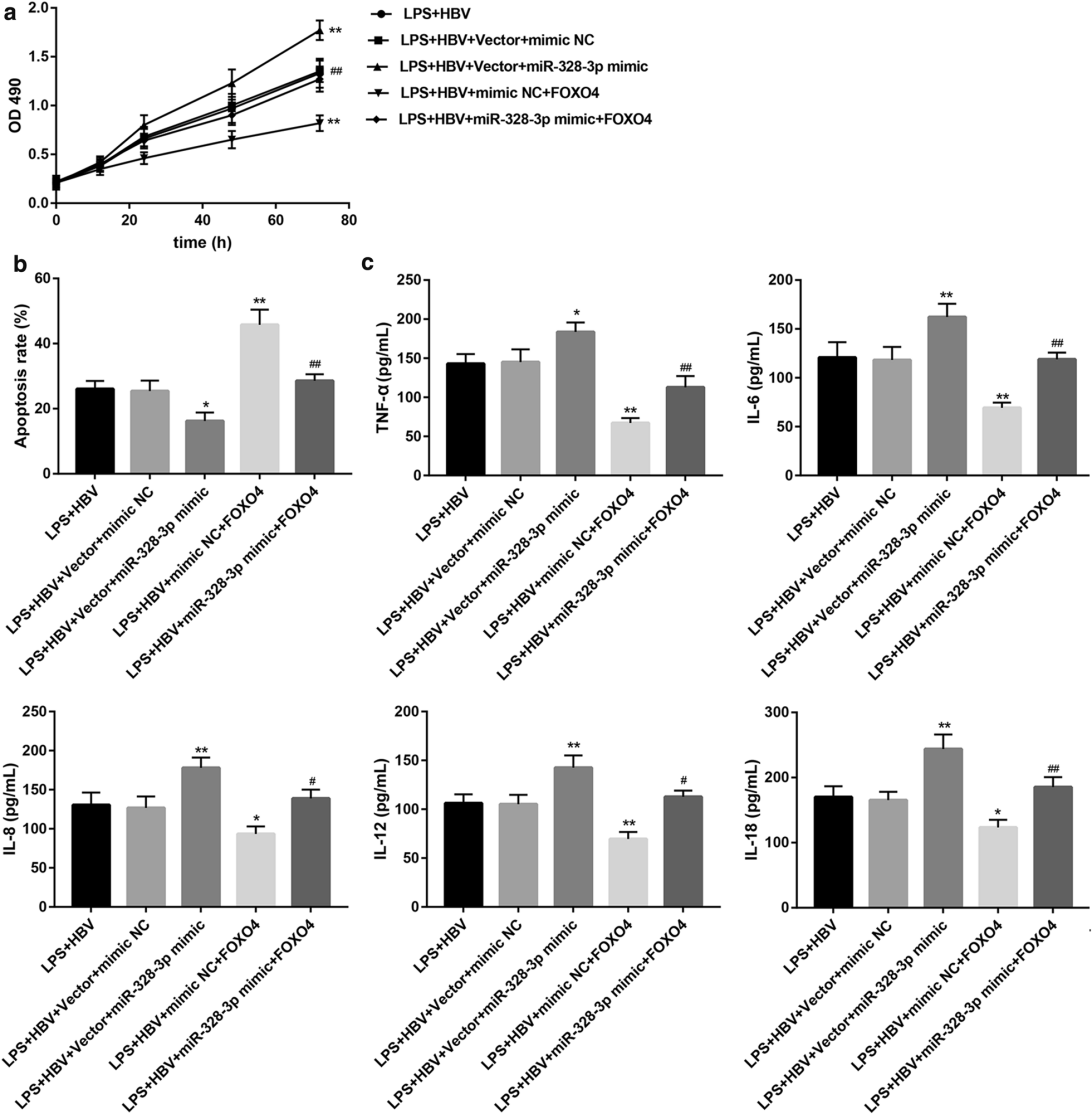
FOXO4 overexpression abrogates the miR-328-3p-mediated cell injury. THLE-2 cells were co-transfected with pHBV1.3 or empty pcDNA3.1 vector (control), and miR-328-3p mimic or mimic negative control (NC), under LPS (1 μg/mL, 24 h) and HBV stimulation. **a** Cell proliferation at different time points was examined by MTT assay. **b** Cell apoptosis was examined by flow cytometry and the apoptosis rate was shown. **c** Levels of TNF-α, IL-6, IL-8, IL-12, and IL-18 were measured by ELISA. Values are presented as the mean ± SD (n = 3). *P < 0.05, **P < 0.01 vs. LPS + HBV + Vector + mimic NC. ^#^P < 0.05, ^##^P < 0.01 vs. LPS + HBV + Vector + miR-328-3p mimic

Finally, we determined whether FOXO4 is involved in miR-328-3p-mediated cell injury under LPS and HBV stimulation. We found that contrary to miR-328-3p inhibitor (Fig. 4), miR-328-3p mimic promoted cell proliferation (Fig. 8a), inhibited cell apoptosis (Fig. 8b and Additional file 3: Figure S3), and induced levels of pro-inflammatory cytokines (Fig. 8c), further indicating that miR-328-3p mediated cell injury. Importantly, FOXO4 overexpression significantly abrogated the miR-328-3p-mediated cell injury under LPS and HBV stimulation (Fig. 8a–c).

## Discussion

The results described above in this study support a HBV-STAT3-miR-328-3p-FOXO4 regulatory cascade in regulating HBV-infected THLE-2 cell injury: (i) HBV increases miR-328-3p expression through a STAT3-mediated activation of miR-328-3p transcription and (ii) HBV-induced miR-328-3p downregulates FOXO4 by targeting its 3′-UTR. The resulting reduced FOXO4 would lead to activation of the NF-κB pathway and cell inflammation and injury. Therefore, we propose that the HBV-STAT3-miR-328-3p-FOXO4 regulation pathway may underlie the pathogenesis related to chronic HBV infection. To our knowledge, the molecular details of STAT3-dependent activation of miR-328-3p by HBV and downregulation of FOXO4 by miR-328-3p in THLE-2 cells are reported here for the first time.

The pathogenesis of HBV-induced hepatitis is complicated. Previous studies have indicated that expression of serum miR-328 is associated with several diseases such as cancer [25, 26], atherosclerosis [27], and immunity-related diseases [28]. Importantly, downregulated miR-328 had an inhibitory effect on cell invasion and growth in hepatocellular carcinoma [29]. To date, multiple targets of miR-328 have been identified [27, 29]. In HBV-related studies, miR-328-3p has been identified by us as a potent predictor for the prognosis of HBV-related ACLF [9]. The results presented here showed that serum miR-328-3p expression was significantly upregulated in both CHB and ACLF patients compared with the normal control subjects. Our in vitro assay revealed that in human liver cell line THLE-2, HBV/HBc/HBx increased miR-328-3p expression. Importantly, miR-328-3p inhibitor significantly suppressed HBV activity and attenuated the HBV-mediated cell injury, whereas miR-328-3p mimic exerted the opposite effect. Therefore, our studies indicate that miR-328-3p plays an important role in the pathogenesis of HBV infection and serve as a potential therapeutic target for HBV infection.

STAT3 has been shown to play important roles in liver inflammatory responses [16]. Previous studies showed that HBV activates STAT3 signaling in hepatocytes to foster its own replication but also to prevent apoptosis of infected cells [14]. Furthermore, knockout of HbsAg inhibits STAT3 signaling, while overexpression of HBsAg induces accumulation of STAT3 phosphorylation [15]. Consistent with these observations, our results showed that HBV/HBc/HBx increased miR-328-3p expression and STAT3 phosphorylation. Furthermore, our bioinformatics analysis (LASAGNA-Search 2.0 and TransmiR v2.0 database) and CHIP assay confirmed the direct binding of STAT3 to the miR-328-3p promoter. Further functional assay revealed that STAT3 activated miR-328-3p transcription and mediated the HBV/HBc/HBx-induced upregulation of miR-328-3p. Therefore, these findings suggest the potential role of the STAT3-miR-328-3p axis in regulating cell injury in HBV-infected THLE-2 cells.

FOXO4 is a member of the Forkhead (Fox) transcription factor O family and was initially identified as a tumor suppressor which limits cell proliferation and induces apoptosis [30]. Interestingly, HBV/HBc/HBx increased miR-328-3p expression but decreased FOXO4 expression. Furthermore, miR-328-3p targeted FOXO4 and decreased FOXO4 protein expression. Moreover, FOXO4 overexpression significantly suppressed HBV activity and attenuated the HBV-mediated cell injury, whereas FOXO4 silencing caused the opposite effect. Importantly, FOXO4 overexpression abrogated the miR-328-3p mimic-mediated cell injury under LPS and HBV stimulation.

The results presented here also demonstrated miR-328 mimic decreased IκB-α protein expression and increased p65 phosphorylation, indicating the activation of the NF-κB pathway. In contrast, miR-328 inhibitor suppressed NF-κB pathway. Researches have shown that FOXO4-knock out (KO) mice exhibits upregulated inflammatory cytokines in colons [19], and mechanistically, FOXO4 interacts with NF-κB and inhibits its DNA binding and transcriptional activity [19]. They also proposed that inflammatory signals activate NF-κB and meanwhile inactivate FoxO4 possibly through interferon regulatory factors, releasing its inhibition of NF-κB to allow maximum activation to combat inflammation [19]. In combination with our results, these findings may indicate the role of the miR-328-3p-FOXO4-NF-κB axis in regulating cell inflammation and injury in HBV-infected THLE-2 cells. However, one limitation of this study was that only one cell line THLE-2 was used. Further studies in other liver cells were needed to confirm our results in this study.

## Conclusion

**Fig. 9:**
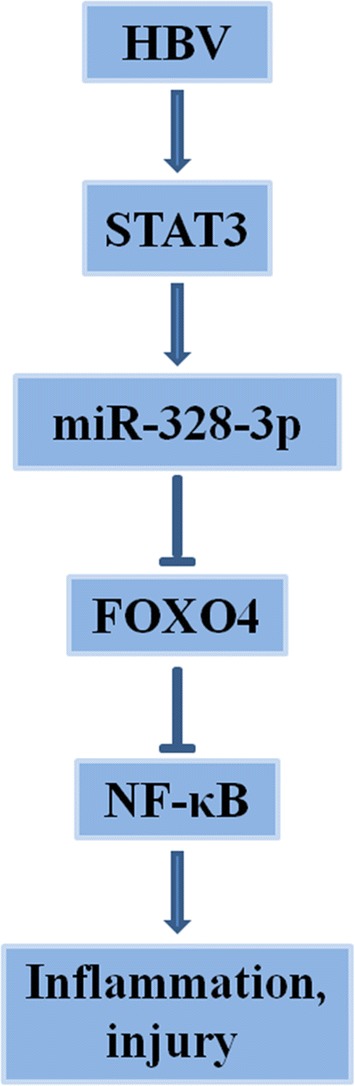
Schematic diagram of the potential roles of miR-328-3p in modulating cell injury in HBV-infected liver cell line THLE-2

In conclusion, our findings demonstrate that HBV upregulates miR-328-3p in HBV-infected THLE-2 cells by promoting the binding of STAT3 to the miR-328-3p promoter. Subsequently, miR-328-3p targets FOXO4 and activates downstream NF-κB pathway, leading to promotion of THLE-2 cell inflammation and injury (Fig. 9). These data demonstrate that HBV-STAT3-miR-328-3p-FOXO4 regulation pathway may play an important role in the pathogenesis of HBV infection. These findings will also be important for identifying potential therapeutic targets for HBV infection.

## Supplementary information

**Additional file 1: Figure S1.** THLE-2 cells were transfected with miR-328-3p inhibitor or inhibitor negative control (NC), under stimulation with LPS (1 μg/mL, 24 h) or/and HBV. Representative scatter plot detecting apoptosis by flow cytometry. The apoptosis rate was shown in Fig. 4b.

**Additional file 2: Figure S2.** THLE-2 cells transfected with pcDNA3.1-FOXO4, si-FOXO4, or corresponding controls, under LPS (1 μg/mL, 24 h) and HBV stimulation. (A) The overexpression and knockdown efficiencies of FOXO4 were confirmed by qRT-PCR. (B) Representative scatter plot detecting apoptosis by flow cytometry. The apoptosis rate was shown in Fig. 6d.

**Additional file 3: Figure S3.** Representative scatter plot detecting apoptosis by flow cytometry. The apoptosis rate was shown in Fig. 8b.

## Data Availability

The datasets used and/or analysed during the current study are available from the corresponding author on reasonable request.

## Abbreviations

3′-UTR: 3′-untranslated region
ACLF: Acute-on-chronic liver failure
ASC: Chronic asymptomatic HBV carriers
ATCC: American Type Culture Collection
CHB: Chronic hepatitis B
CHIP: Chromatin immunoprecipitation
DMEM: Dulbecco’s modified Eagles medium
FBS: Fetal bovine serum
FITC: Fluorescein isothiocyanate
HBcAg: Hepatitis B virus core antigen
HBp: Hepatitis B virus DNA polymerase protein
HBsAg: Hepatitis B virus surface antigen
HBV: Hepatitis B virus
HBx: Hepatitis B virus X protein
HRP: Horseradish peroxidase
IL-12: Interleukin-12
IL-18: Interleukin-18
IL-6: Interleukin-6
IL-8: Interleukin-8
KO: Knock out
miRNAs: MicroRNAs
MTT: 3-4,5-Dimethylthiazol-2-yl -2,5-diphenyltetrazolium bromide
MUT: Mutated
NC: Normal controls
OD: Optical density
PI: Propidium iodide
PVDF: Polyvinylidene fluoride
qRT-PCR: Real-time quantitative PCR
SDS-PAGE: Sodium dodecyl sulfate–polyacrylamide gel electrophoresis
STAT3: Signal transducer and activator of transcription 3
TNF-α: Tumor necrosis factor-alpha
WT: Wild-type
