# Supplementary figures and images for: Upregulation of microRNA-328-3p by hepatitis B virus contributes to THLE-2 cell injury by downregulating FOXO4
Source: J Transl Med. 2020 Mar 30;18:143. doi: 10.1186/s12967-020-02299-8 (PMC7106851; doi:10.1186/s12967-020-02299-8)

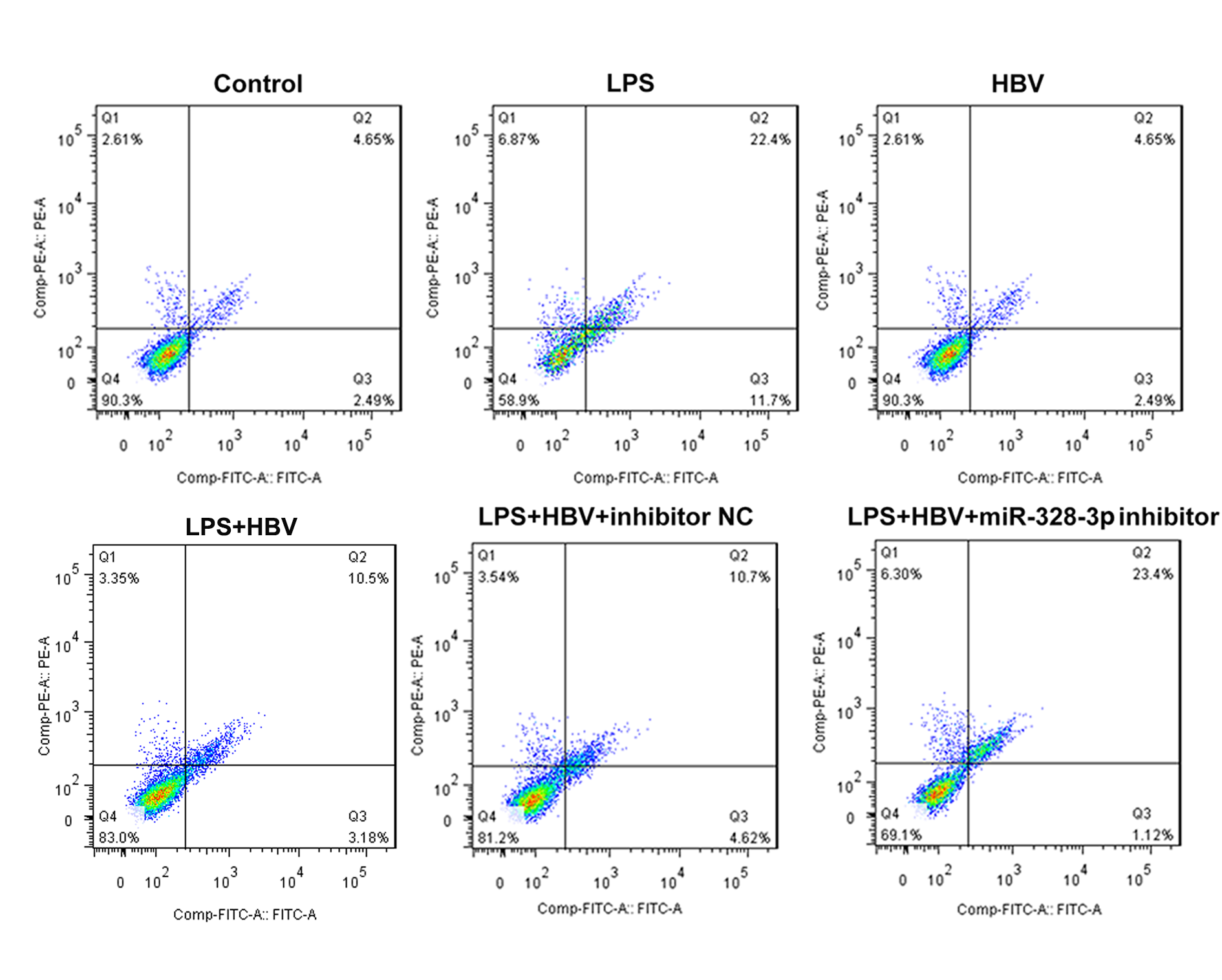

Supplement: Supplementary file 1 — Additional file 1: Figure S1. THLE-2 cells were transfected with miR-328-3p inhibitor or inhibitor negative control (NC), under stimulation with LPS (1 μg/mL, 24 h) or/and HBV. Representative scatter plot detecting apoptosis by flow cytometry. The apoptosis rate was shown in Fig. 4b. [file 12967_2020_2299_MOESM1_ESM.tif]

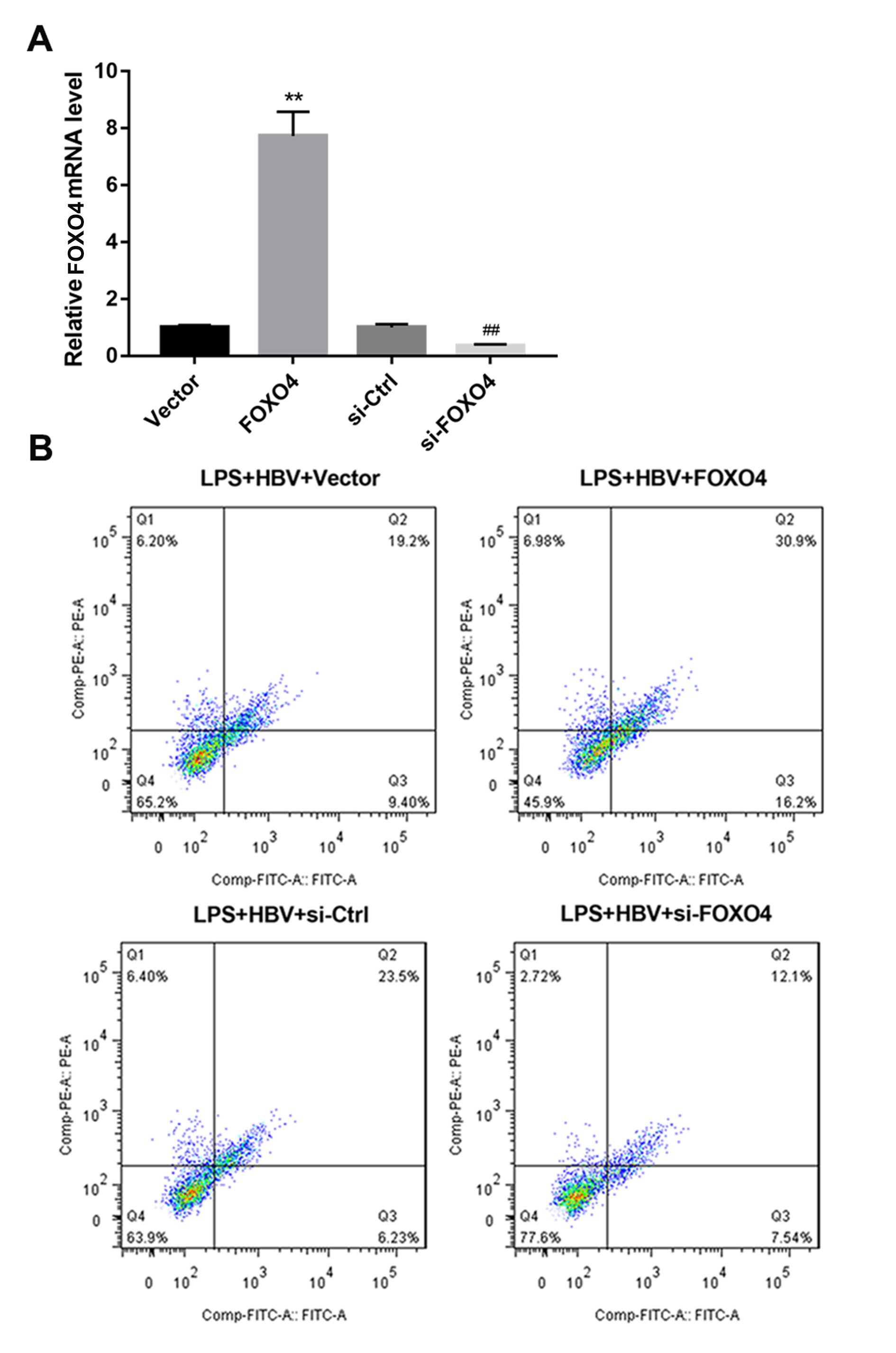

Supplement: Supplementary file 2 — Additional file 2: Figure S2. THLE-2 cells transfected with pcDNA3.1-FOXO4, si-FOXO4, or corresponding controls, under LPS (1 μg/mL, 24 h) and HBV stimulation. (A) The overexpression and knockdown efficiencies of FOXO4 were confirmed by qRT-PCR. (B) Representative scatter plot detecting apoptosis by flow cytometry. The apoptosis rate was shown in Fig. 6d. [file 12967_2020_2299_MOESM2_ESM.tif]

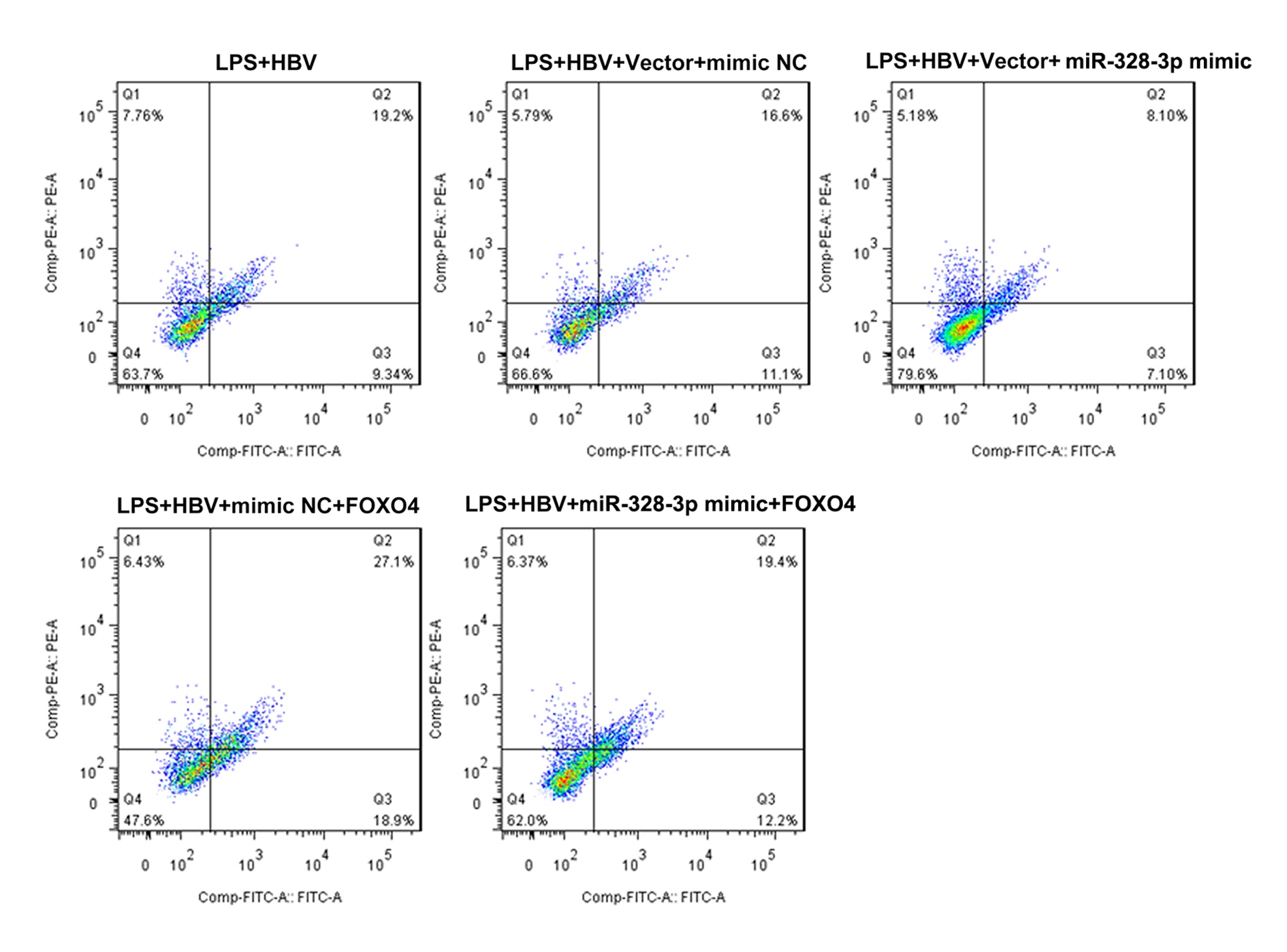

Supplement: Supplementary file 3 — Additional file 3: Figure S3. Representative scatter plot detecting apoptosis by flow cytometry. The apoptosis rate was shown in Fig. 8b. [file 12967_2020_2299_MOESM3_ESM.tif]
